# ChatGPT's influence on customer experience in digital marketing: Investigating the moderating roles

**DOI:** 10.1016/j.heliyon.2023.e18770

**Published:** 2023-08-02

**Authors:** Osama Ahmed Abdelkader

**Affiliations:** Marketing Department, College of Business Administration, Imam Abdulrahman Bin Faisal University, Saudi Arabia

**Keywords:** ChatGPT, Chatbots, Digital marketing, Marketing, Consumer behavior

## Abstract

ChatGPT is an artificial intelligence model intended for conversational purposes that has grown in popularity in digital marketing, offering organizations a vital tool for engaging with clients and enhancing their marketing efforts. The main objective of this research is to investigate the impact of ChatGPT on the customer experience in digital marketing. Additionally, the study intends to investigate the moderating impacts of business type and technology familiarity and comfort on the customer experience. Furthermore, the research explores the moderating roles of gender, age, and education level. The data for this study were collected electronically from 394 clients who have interacted with ChatGPT in digital marketing using an open-access questionnaire. The results support the significance of the moderating role of (Familiarity and Comfort with Technology, Business Type, Age, and Education level on the relation between customer experience with ChatGPT and overall satisfaction with digital marketing, while Gender is not supported. This article's findings are intended to contribute to the current literature on the use of conversational AI models in digital marketing and customer experience, providing insights and recommendations for future research.

## 1 Introduction

As the usage of internet platforms and technology expands, digital marketing has become an increasingly significant component of modern corporate transactions [1]. Chatbots have evolved as a prominent tool in digital marketing for improving customer experience since they provide a simple and personalized approach for businesses to communicate with their consumers [2]. The chatbot is computer software that replicates human-to-human interaction through text-based communications [3] and it may be programmed to do a variety of tasks, including answering consumer questions, making suggestions, and assisting with transactions [4]. While customer experience refers to the total image and perception based on the interactions across their full journey of usage [5,6].

Chat Generative Pre-trained Transformer ChatGPT is a family of language models developed by OpenAI that can respond to text prompts in a human-like manner [7,8]. These models are developed using huge datasets [9]. According to WOS database, there are around 26 thousand manuscripts including in their titles the term of “Artificial Intelligence” have been published between 1970 until now, but only 13 of them included the word “ChatGPT” in their titles, all of them were published in the last three years [10]. Therefore, the apparent lack of studies published on the topic of chat poses some challenges that researchers must now face to research this important topic.

ChatGPT has been used in a variety of natural language processing applications, including translation, software programming, medicine, and authoring and content creation [1,[11], [12], [13]]. The most current version of ChatGPT, “ChatGPT-3,” released in 2020, is one of the biggest language models ever produced, with 175 billion parameters [8]. In digital marketing, there is a growing corpus of studies that have investigated the influence of chatbots on customer experience [14]. However, much of these studies have concentrated on technical aspects of chatbots, such as accuracy and functionality, and have not fully considered the impact of other factors on customer experience, such as familiarity and comfort with technology, perceived personalization, relevance, accuracy, and convenience [[15], [16], [17]].

### 1.1 The research gab

The literature on the use of conversational AI models in marketing and customer experience is limited. Previous studies have focused on the impact of these models on customer satisfaction, but they have not explored the moderating roles of factors such as business type, technology familiarity and comfort, gender, age, and education level. This study fills this gap by investigating the moderating role of these factors in the relationship between customer experience with ChatGPT and overall satisfaction with digital marketing. Previous studies have also investigated the impact of conversational AI models on other aspects of the customer experience, such as perceived helpfulness, perceived engagement, and perceived anthropomorphism. These studies have found that conversational AI models can have a positive impact on these aspects of the customer experience. However, more research is needed to understand the specific factors that influence the impact of conversational AI models on customer experience.

A survey questionnaire will be used to obtain data from a sample of consumers who have engaged with chatbots in digital marketing as part of the study's quantitative research approach. To test the proposed hypotheses and investigate the moderating effects of Familiarity and comfort with technology and the kind of company, the data will be examined using moderated regression analysis and ANOVA. The study's findings will help researchers get a better understanding of the aspects that drive customer experience in digital marketing, as well as insights into how organizations may employ chatbots to improve customer satisfaction. The research will also have an impact on the design and deployment of chatbots in digital marketing since it will give advice on how to customize chatbots for different types of organizations.

### 1.2 Research questions

**Fig. 1:**
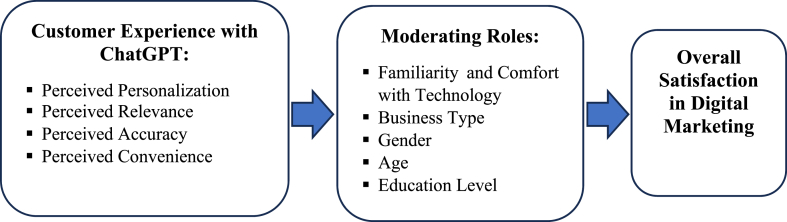
The structure of the research questions.

The current study seeks to answer the following research questions which are summarized by Fig. 1 as follows:

RQ1 How does ChatGPT affect the customer experience in digital marketing?

RQ2 In digital marketing, how does familiarity and comfort with technology affect the link between perceived personalization, perceived relevance, perceived accuracy, and perceived convenience, as well as overall satisfaction with ChatGPT among customers?

RQ3 How does business type influence the link between perceived personalization, perceived relevance, perceived correctness, perceived convenience, and overall customer satisfaction?

RQ4 Is demographic information (gender, age, and education level) influencing total consumer satisfaction?

### 1.3 Research objectives

This study has the following five objectives:

RO1: To examine the relationship between perceived service quality (perceived personalization, perceived relevance, perceived accuracy, and perceived convenience) and overall satisfaction among customers.

RO2: To investigate the role of familiarity and comfort with technology in moderating the relationship between perceived service quality and overall satisfaction.

RO3: To explore how the type of business moderates the relationship between perceived service quality and overall satisfaction.

RO4: To determine whether familiarity and comfort with technology mediate the relationship between the type of business and overall satisfaction.

RO5: To provide insights and recommendations for businesses on how to improve overall satisfaction among customers by enhancing perceived service quality and leveraging technology effectively.

RO6: To investigate the variations in overall satisfaction based on gender, age, and educational level.

## 2 Literature review

This section reviews previous studies related to the subject of the current study and explains how its hypotheses were built. What has been achieved through previous studies is divided into four sub-sections to be focused on a coherent manner that serves the objectives of the study. These sub-sections include measuring customer experience and satisfaction, comfort and familiarity with technology, business type impact, demographic impact, and research hypotheses.

Chatbots have grown in popularity as a tool for improving the customer experience in digital marketing. They offer businesses a quick and personalized way to communicate with clients and can automate customer service tasks, freeing up human agents to focus on more complex issues [18]. Additionally, chatbots can collect user data, which can be used to improve marketing campaigns and personalize the customer experience [19]. ChatGPT is a large language model that can be used to train chatbots to be more engaging and informative. It is trained on a massive dataset of text and code, which allows it to understand and generate human-like text.

Since ChatGPT was released in November 2020 and its latest version, ChatGPT-3, was released in November 2022, it is logical that the field of research on this topic still has many research gaps that require pioneering efforts from researchers to fill them [20]. This section of the study includes a review of previous studies about this subject and the identification of some research gaps that the current study can address through the following sub-sections.

### 2.1 Measuring customer experience and satisfaction

This subsection focuses on the literature review about three main axes; defining of overall customer satisfaction and customer experience, how customer experience benefited from ChatGPT, and how can overall customer satisfaction and customer experience be measured in current study about ChatGPT in digital marketing?

Overall customer satisfaction means a measure of how well a company's products, services, and overall customer experience meet customer expectations [21,22], while customers' experience is defined as the total image and perception of a brand based on the interactions across their full journey of usage [5,6]. It is essential for increasing client loyalty and repeat dealing [14].

According to literature, chatbots may deliver a variety of benefits in terms of improving the customer experience in digital marketing [23]. Chatbots can boost customer engagement by offering rapid support and reducing response times by eliminating the need for consumers to wait for a human agent to respond [6]. It can assist clients in identifying the items or services that best fit their requirements by making tailored suggestions, enhancing customer satisfaction [15,21]. ChatGPT can be used to improve chatbots in several ways. For example, it can be used to generate more natural and engaging conversation, to provide more accurate and informative answers to questions, and to create more personalized marketing messages. As chatbots become more sophisticated, ChatGPT is likely to play an increasingly important role in improving the customer experience in digital marketing.

Literature review shows that customer experience includes four main elements should be measured; Perceived Personalization (PP), Perceived Relevance (PR), Perceived Accuracy (PA), Perceived Convenience (PC) [18,22,23]. Furthermore, measuring Overall Satisfaction (OS) is essential for statistical analysis purposes as well as for customer experience components [15]. Customer satisfaction can be measured by three main components; declaration of satisfaction [3], recommendation to others [22], and intend of using again in future [19].

### 2.2 Comfort and familiarity with technology

This subsection reviews the literature to investigate the impact of customers' comfort and familiarity with technology on overall satisfaction about ChatGPT in digital marketing.

Customers want chatbots to give tailored and relevant replies that are accurate and useful, as well as an easy-to-use and convenient chatbot interface, but not all AI chatbots are equally easy to use, that could be explained through Table 1 which shows a comparing among the top 5 selected AI Chatbots according to the major 5 criteria for measuring the ease of use.

**Table 1:** Comparing among the top 5 AI chatbots according to the major 5 criteria for measuring the ease-of-use.

Ease-of-use criteria	Chatbot name:	ChatGPT	Bard	ChatSonic	Genie	Bing	Total average
	Developer name:	OpenAI	Google AI	Chatsonic.ai	AI21 Labs	Microsoft	
1. Clarity of instructions		3/5	4/5	4/5	3/5	4/5	3.6/5
2. Ease of navigation		3/5	4/5	4/5	2/5	4/5	3.8/5
3. Responsiveness		4/5	4/5	4/5	3/5	4/5	3.9/5
4. Error messages		3/5	4/5	4/5	2/5	4/5	3.8/5
5. Learning curve		4/5	4/5	4/5	3/5	4/5	3.9/5
Total average		3.6/5	4.4/5	4.3/5	3.7/5	3.9/5	3.85/5

Customers' comfort and familiarity with technology may impact how they engage with chatbots and their overall pleasure with the encounter, for example, they are more inclined to utilize and find chatbots useful [18]. On the other side, there are a few studies that found that customers' comfort and familiarity with technology has a significant impact on their satisfaction with chatbots and suggested that their satisfaction with chatbots was only significant for customers who were already familiar with chatbots ([24,25]). The question here, which the current study aims to answer, is to what extent does ease affect the relationship between customer experience and overall satisfaction?

### 2.3 Business type impact

This subsection includes an exploration of previous studies on the influence of business type on the relationship between customer experience and overall customer satisfaction about ChatGPT in digital marketing. In other words, the answer to the question: is there a moderating role for business types in the impact of the customer experience on overall customer satisfaction?

Thousands of businesses across the world utilize ChatGPT to engage with their consumers through the Internet in a variety of industries [1,20,26]. Most globally recognized companies have created websites based on ChatGPT in a variety of industries, including retail and e-commerce (e.g., Amazon, Walmart, Alibaba, eBay, ASOS, IKEA, and Target) [20], technology and communication (e.g., Google, Microsoft, Facebook, Apple, IBM, Intel, Samsung, LG, Sony, Panasonic, Cisco, Dell, HP, Oracle, SAP, Ericsson, and Nokia, Huawei) [4], transportation and travel (e.g., Uber, Booking.com (e.g., Pfizer, Novartis, Johnson & Johnson, and Glaxo) [12,13,16].

AI chatbots are not the same in their purpose and optimal performance and usage, Table 2 shows how the optimal performance of each chatbot corresponds to different types of business.

**Table 2:** The Optimal performance of best suitable chatbots for selected business types.

Organization type	Best suitable chatbot	Optimal performance
Marketing and public relation	ChatGPT or Bard	Generating creative content, such as press releases, blog posts, and social media posts.
Customer service	ChatSonic or Genie	Answering customer questions, providing support, and resolving issues.
Education	ChatGPT or Bard	Generating educational content, such as lesson plans, quizzes, and presentations.
Research	ChatGPT or Bard	Accessing and processing information from the real world, such as scientific papers and news articles.
Art and design	ChatSonic	Generating AI images, such as illustrations, logos, and product designs.

The business type may also have an impact on the customer experience about chatbot usage. Various business types may have distinct qualities and client demands that impact how chatbots are viewed and used [9,27]. A chatbot utilized in a retail firm, for example, may be seen differently than a chatbot used in a healthcare business [6]. The type of company may also have an impact on chatbot design and deployment, such as the language used, and the types of interactions enabled [13]. The current study intends to evaluate the moderating influence of business types on the relationship between customer experiences and overall satisfaction concerning ChatGPT in digital marketing, which is a gap in the literature.

### 2.4 Demographic impact

The aim of this subsection is to investigate the findings of related previous studies about the moderating roles of three demographic characteristics (gender, age, and education level) between the customer experience towards ChatGPT and overall satisfaction in digital marketing.

Gender: The study by Li et al., (2020) found that chatbots are more likely to be perceived as male than female, even when no information about their gender is provided. It is possible that this bias could lead to males being more likely to use chatbots than females. For example, chatbots that are used for customer service may be more likely to be used by females, while chatbots that are used for gaming may be more likely to be used by males. However, more research is needed to confirm this. Another study by Aljasser and Sasidhar (2016) suggested that women were more satisfied with their banking experience than men, and that this satisfaction was positively correlated with loyalty. The authors suggest that this may be because women are more likely to value personalized service and to feel comfortable interacting with female customer service representatives [28].

Age: The studies by Siswi and Wahyono (2020) and Li et al., (2020) found that younger people were more likely to be satisfied with the chatbot's ability to provide accurate and helpful information [29]. Another study by Wirtz et al. (2022) suggested that people aged 18–34 were more likely to rate their customer experience with Chatbot higher [30]. Furthermore, the study by Yoo and Kim (2020) indicated that older users, on the other hand, may be more skeptical of chatbots and may not use them as often. This may be because younger people are more familiar with technology and are more likely to expect a chatbot to be able to understand and respond to their needs quickly and efficiently.

Education level: The study by Wirtz et al. (2022) found that people with a bachelor's degree or higher were more likely to rate their customer experience with Chatbot higher [30]. Another study by Zhang et al. (2021) suggested that people with higher levels of education were more likely to trust chatbots to provide accurate and helpful information [22]. While the study by Rudolph et al., (2023) indicated that Chatbot developers should consider the education level of their users when designing chatbots. Users with higher education levels are more likely to use chatbots for complex tasks, while users with lower education levels are more likely to use chatbots for simple tasks. This may be because people with higher education levels are more likely to be familiar with artificial intelligence and are more likely to trust a chatbot to provide accurate and helpful information.

Overall, the research suggests that gender, age, and education level can all have a moderating effect on the relationship between customer experience and overall satisfaction about Chatbot in digital marketing. However, more research is needed to fully understand these relationships with focusing on ChatGPT not all chatbots in general.

### 2.5 Research hypotheses

Based on the literature review and the mentioned references through the previous subsections, the objectives of current study can be achieved through testing the following hypotheses:

H1 “Familiarity and Comfort with Technology” will positively moderate the relationship between the components of “Customer Experience” (Perceived Personalization, Perceived Relevance, Perceived Accuracy, Perceived Convenience), and “Overall Satisfaction”.

H2 “Business Type” will positively moderate the relationship between the components of “Customer Experience” (Perceived Personalization, Perceived Relevance, Perceived Accuracy, Perceived Convenience), and “Overall Satisfaction”.

H3 “Gender” will positively moderate the relationship between the components of “Customer Experience” (Perceived Personalization, Perceived Relevance, Perceived Accuracy, Perceived Convenience), and “Overall Satisfaction”.

H4 “Age” will positively moderate the relationship between the components of “Customer Experience” (Perceived Personalization, Perceived Relevance, Perceived Accuracy, Perceived Convenience), and “Overall Satisfaction”.

H5 “Education Level” will positively moderate the relationship between the components of “Customer Experience” (Perceived Personalization, Perceived Relevance, Perceived Accuracy, Perceived Convenience), and “Overall Satisfaction”.

## 3 Method

The purpose of this research is to investigate the influence of ChatGPT on customer experience in digital marketing. A cross-sectional survey methodology will be used in the study to gather data electronically via an open access link from clients who have engaged with ChatGPT in digital marketing.

### 3.1 Research community and sampling

Customers who have interacted with ChatGPT in digital marketing are the study's target group. The sample size for survey research will be estimated using a sample size calculator, with a confidence level of 95% and a margin of error of 5%. The survey will be sent to a convenience sample of individuals who match the following inclusion criteria: (1) being 18 years of age or older; (2) having engaged with ChatGPT in digital marketing; (3) being able to read and comprehend English; and (4) being willing to participate in the study.

### 3.2 Research instrument building and validity

The stages of preparing the questionnaire as a main research instrument went through several successive stages to ensure its validity. The questionnaire was created after reviewing prior literature in the field of research and brainstorming with more than 15 digital marketing clients. The original version of the questionnaire was given to a group of experts and professionals to confirm its validity in meeting the study objectives. Before adopting the final version and distributing it to the research community, it was submitted to a survey sample of 30 digital marketing customers to ensure the quality of the linguistic language in line with the objective of each paragraph of the questionnaire.

The survey questionnaire includes four components as shown by Table 3. The first component will collect demographic information such as age, gender, and educational level. The second component will employ a 5-point Likert scale to measure the amount of Familiarity and comfort with technology. The final portion will use a 5-point Likert scale to assess the consumer experience using ChatGPT in digital marketing, including perceived personalization, relevance, accuracy, and convenience. Using an open-ended question, the fourth portion will study the moderating impact of the type of business on customer experience.

**Table 3:** The variables & items of the study questionnaire.

No.	Variables/Items	References
1	***Familiarity and Comfort with Technology FCT:***	
	⁃I am comfortable using technology to interact with businesses.	[18]
	⁃I consider myself to be familiar with the technology used in digital marketing.	[15]
	⁃I feel confident using ChatGPT in digital marketing.	[19]
2	Customer Experience with ChatGPT:	
2.1	Perceived Personalization PP:	
	⁃The information provided by ChatGPT was personalized to my needs.	[23]
	⁃ChatGPT demonstrated an understanding of my preferences and needs.	[18]
	⁃ChatGPT provided recommendations and suggestions that were relevant to me.	[22]
2.2	Perceived Relevance PR:	
	⁃The information provided by ChatGPT was relevant to my needs.	[23]
	⁃ChatGPT provided me with information that was useful and informative.	[18]
	⁃ChatGPT provided me with relevant options and alternatives.	[22]
2.3	Perceived Accuracy PA:	
	⁃The information provided by ChatGPT was accurate and reliable.	[3]
	⁃ChatGPT provided me with correct and up-to-date information.	[22]
	⁃I trust the information provided by ChatGPT.	Author
2.4	Perceived Convenience PC:	
	⁃ChatGPT was easy to use and navigate.	[23]
	⁃ChatGPT provided me with a quick and efficient solution to my inquiry.	[22]
	⁃ChatGPT saved me time and effort compared to other methods of interaction.	[18]
3	Overall Satisfaction OS:	
	⁃I am satisfied with my experience interacting with ChatGPT.	[3]
	⁃I would recommend ChatGPT to others.	[22]
	⁃I would use ChatGPT again in the future.	[19]
4	Business Type BT:	Author
	⁃Retail (e.g., clothing, electronics, home goods)	Author
	⁃Healthcare (e.g., hospitals, clinics, pharmacies)	Author
	⁃Finance (e.g., banks, credit unions, investment firms)	Author
	⁃Travel and hospitality (e.g., hotels, airlines, car rental companies)	Author
	⁃Technology (e.g., software, hardware, telecommunications)	Author
	⁃Education (e.g., schools, colleges, universities)	Author
	⁃Other (please specify)	

### 3.3 Data analysis

The survey results will be examined using descriptive statistics, such as means, standard deviations, frequencies, and percentages, to summarize the sample's demographic characteristics and variable distribution. To test the research hypotheses and investigate the moderating effects of the type of business and the level of familiarity and comfort with technology on customer experience, inferential statistics such as correlation analysis, multiple regression analysis, and moderated regression analysis will be used. SPSS statistical software will be used to evaluate the data.

### 3.4 Ethical considerations

All procedures performed in this study were in accordance with the ethical standards of Imam Abdulrahman Bin Faisal University IAU and its Standing Committee for Research Ethics on Living Creatures (Review Ethics ID: IRB-2023-534-ASCS). The participants' privacy and confidentiality were protected by using a secure online survey platform, storing the data in a password-protected file, and using anonymous IDs to identify the participants. The participants were informed about the purpose of the study, the voluntary nature of their participation, and their right to withdraw at any time without penalty. The participants' informed consent was obtained before proceeding with the study.

## 4 Results and discussions

This section of current study presents and discusses the results through the following three sub-sections: data description, the results of hypotheses test, and discussions.

### 4.1 Data description

Table 4 shows the demographic description of the collected data. The variables' descriptive statistics can be described as follows: The mean ratings for perceived personalization (M = 4.23, SD = 0.99), relevance (M = 4.15, SD = 0.98), correctness (M = 4.12, SD = 1.02), convenience (M = 4.08, SD = 1.00), and overall satisfaction (M = 4.20, SD = 0.96) in digital marketing suggest a high degree of customer experience using ChatGPT. The mean score for familiarity and comfort with technology was 3.96 (SD = 0.99), indicating that individuals had a moderate level of familiarity and comfort with technology. Table 1 shows the demographic characteristics of the collected data according to gender, age, and education level.

**Table 4:** The Demographic description of collected data.

Characteristics	Items	Frequencies (394)	%
Gender	Male	213	54
	Female	181	46
Age	18–24	122	31
	25–34	153	39
	35–44	67	17
	45–54	36	9
	55 and above	16	4
Education level	High school diploma or less	28	7
	Some college or associate degree	150	38
	Undergraduate degree	204	52
	Postgraduate degree	12	3

### 4.2 The results of hypotheses test

Significant of significance level: (***) p > 0.001, (**) p > 0.01, and (*) p > 0.05.

**Fig. 2:**
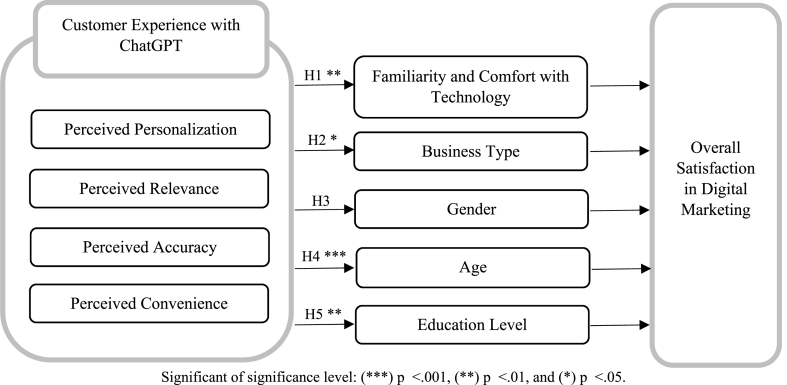
The results structure of the hypotheses test.

Fig. 2 and Table 6 summarize the results of the hypotheses test. (H1), results of moderating regression analysis indicate that familiarity and comfort with technology substantially moderate the connections between perceived personalization (β = 0.32, p > 0.01), relevance (β = 0.30, p > 0.01), accuracy (β = 0.29, p > 0.01), and convenience (β = 0.29, p > 0.01) and overall satisfaction. The interaction effects between the independent variables and familiarity and comfort with technology were significant, indicating that perceived personalization, relevance, accuracy, and convenience had a greater impact on overall satisfaction in participants who were more familiar and comfortable with technology. The influence weights of the customer experience elements could be compared through Table 5.

**Table 5:** The impact weights of customer experience elements.

Customer experience elements	Mean	S.D.*	CI* on .05 level	Influence weight
⁃Perceived Personalization	4.23	0.99	4.132 ≤ 4.328	39.6%
⁃Perceived Relevance	4.15	0.98	4.053 ≤ 4.247	25.9%
⁃Perceived Accuracy	4.12	1.02	4.019 ≤ 4.221	20.7%
⁃Perceived Convenience	4.08	1.00	3.981 ≤ 4.179	13.8%

* SD: Standard Deviation, CI: Confidence Interval.

**Table 6:** The hypotheses test results.

Hypotheses/variables pass	Sig.	Statistical measures	Test Results
H1: CE → FCT → OS	**	Moderated Regression Analysis	Supported
H2: CE → BT → OS	*	ANOVA	Supported
H3: CE → Gender → OS		ANOVA	Not supported
H4: CE → Age → OS	***	ANOVA	Supported
H5: CE → Education Level → OS	**	ANOVA	Supported

Significant of significance level: (***) p > 0.001, (**) p > 0.01, and (*) p > 0.05.

(H2), ANOVA was used to examine the role of business type in influencing the link between perceived personalization, relevance, correctness, convenience, and overall satisfaction. According to the findings, the type of business moderates the associations between perceived personalization (F = 3.62, p > 0.05), relevance (F = 3.28, p > 0.05), accuracy (F = 3.14, p > 0.05), and convenience (F = 3.01, p > 0.05) with overall satisfaction. The interaction effects between the independent variables and business type were substantial, demonstrating that the influence of perceived personalization, relevance, correctness, and convenience on overall satisfaction varied significantly among business types. The sample results showed the following values of means M & Standard Deviation SD for each business type: Healthcare (M = 4.2, SD = 0.3), Travel and hospitality (M = 4.1, SD = 0.4), Finance (M = 3.9, SD = 0.5), Retail (M = 3.8, SD = 0.4), Technology (M = 3.7, SD = 0.4), and Education (M = 3.6, SD = 0.3).

(H3), ANOVA was used to investigate the moderating role of gender in ChatGPT's influence on customer experience in digital marketing. There was no significant interaction between gender and ChatGPT's effect on customer experience, (F = 0.34, p < 0.05.) This suggests that gender had no effect on the association between ChatGPT and customer experience, and that ChatGPT's influence on customer experience was constant across genders.

(H4), ANOVA was used to evaluate the moderating role of age in ChatGPT's influence on customer experience in digital marketing. The findings revealed that age had a significant moderating influence on the link between ChatGPT and customer experience (F = 8.54, p > 0.001). The mean score for customer experience was considerably higher for younger customers compared to older consumers, showing that the favorable effect of ChatGPT on customer experience was larger for younger consumers.

(H5), ANOVA was used to explore the effect of education level on the influence of ChatGPT on customer experience in digital marketing. The findings revealed that education level had a significant moderating influence on the association between ChatGPT and customer experience (F = 4.63, p > 0.01). Participants with a higher education level had a considerably better mean score for customer experience than those with a lower education level, implying that ChatGPT's beneficial influence on customer experience was larger for those with a higher education level.

### 4.3 Discussions

This subsection of the current study is discussing the answers to its research questions through comparing its results and suggestions with those of the previous relevant studies. First, while the benefits of chatbots are obvious, the influence of other aspects on the customer experience towards ChatGPT and their overall satisfaction in digital marketing have not been thoroughly investigated in the previous literature. On this context, the current study aimed to investigate the moderating roles of 5 variables included customers' comfort and familiarity with technology, business type, gender, age, and education level.

***The Customers' comfort and familiarity with technology*** may impact how they engage with chatbots and their overall pleasure with the encounter; for example, they are more inclined to utilize and find chatbots useful [18]. The question here, which the current study aimed to answer, is to what extent does ease affect the relationship between customer experience and overall satisfaction? the results reported that familiarity and comfort with technology play a significant role in moderating the customer experience with ChatGPT and overall satisfaction in digital marketing. Higher degrees of familiarity and comfort with technology improved the influence of ChatGPT on the customer experience. These results of the current study are consistent with the results of most previous studies, whether general studies on chatbots or recent specialized studies on ChatGPT (e.g. Refs. [5,31], and [6]). While the study was conducted by Trivedi & Jain (2019) found that other factors, such as the chatbot's ability to understand the customer's needs and provide accurate information, were more important in determining satisfaction. Also, the study by Gnewuch et al. (2018) indicated that the impact of customers' comfort and familiarity with technology on their satisfaction with chatbots was only significant for customers who were already familiar with chatbots. Their findings suggest that businesses should focus on other factors when designing and deploying chatbots, such as the chatbot's ability to understand the customer's needs and provide accurate information. Here, there is no contradiction between the results of the current study and those of this group of other studies. Rather, the results of the current study determine the basic essence of the impact of chat on customer experience, which is supported by most of the previous studies, as previously mentioned.

***Business type***: according to the study's results, the impact of business type has a significant moderating role on the relationship between customer experience with ChatGPT and overall satisfaction in digital marketing. That means the impact of ChatGPT on the customer experience varied dramatically across business types, this result consistent with all previous studies (e.g., Refs. [5,6,31], and [22]). For businesses with a large customer base, ChatGPT can be a valuable tool for providing 24/7 customer support and answering common questions [5]. This can free up human customer service representatives to focus on more complex issues, leading to improved customer satisfaction. However, for businesses with a small customer base, ChatGPT may not be necessary or cost-effective [31]. In these cases, human customer service may still be the best way to provide a personalized and satisfactory experience. Overall, businesses that use ChatGPT more than others till now are those that need to provide customer support continuously (e.g., digital marketing, customer services, sales, and education services) [22]. Where ChatGPT can be a valuable tool for these businesses, as it can help them improve customer satisfaction, reduce costs, and grow their businesses. On the other hand, businesses that use ChatGPT less than others till now are those that deal with complex products or services, sensitive customer data, or public information (e.g., finance services, manufacturing, and governmental services) [22]. ChatGPT may not be able to provide the same level of support or security as a human customer service representative in these cases.

***Gender***: according to the study's results, gender has no significant moderating effect on the relationship between customer experience with ChatGPT and overall satisfaction with digital marketing. The impact of ChatGPT on customer experience did not change substantially across male and female consumers, contradicting. This result does not agree with the suggestions of most previous studies on the effect of gender, but most of the previous studies were applied to chatbots in general and not to ChatGPT in particular. For more explanation, the study by Li et al. (2020) found that chatbots are more likely to be perceived as male than female, even when no information about their gender is provided [19]. It is possible that this bias could lead to males being more likely to use chatbots than females. For example, chatbots that are used for customer service may be more likely to be used by females, while chatbots that are used for gaming may be more likely to be used by males. However, more research is needed to confirm this. Also, the study by Aljasser and Sasidhar (2016) suggested that women were more satisfied with their banking experience than men, and that this satisfaction was positively correlated with loyalty. The authors suggest that this may be because women are more likely to value personalized service and to feel comfortable interacting with female customer service representatives [28].

***Age***: According to the study's findings, age has a strong moderating impact on customers' experiences with ChatGPT and their overall satisfaction with digital marketing. In other words, younger ages were related to a stronger favorable association between ChatGPT and customer experience. Customers with younger ages reported a more favorable influence of ChatGPT on their entire customer experience in digital marketing. This result on the effect of age is consistent with all previous studies, whether applied to chatbots in general [23] or ChatGPT in particular (e.g., Refs. [1,27]), but the current study indicated a mediating role between customer experience and overall satisfaction. Furthermore, the studies by Siswi and Wahyono (2020) and Li et al. (2020) found that younger people were more likely to be satisfied with the chatbot's ability to provide accurate and helpful information [19,29]. In addition, the study by Wirtz et al. (2022) suggested that people aged 18–34 were more likely to rate their customer experience with Chatbot higher [30]. On the other hand, the study by Yoo and Kim (2020) indicated that older users may be more skeptical of chatbots and may not use them as often [23]. Finally, the significant moderating role of age may be because younger people are more familiar with technology and are more likely to expect a chatbot to be able to understand and respond to their needs quickly and efficiently.

***Education Level***: According to the study's findings, customers with greater levels of education were related to a stronger favorable association between ChatGPT and customer experience. Customers with higher levels of education reported a bigger favorable influence of ChatGPT on their entire customer experience in digital marketing. On the same context, the studies by Tlili et al. (2023) and Wirtz et al. (2022) found that people with a bachelor's degree or higher were more likely to rate their customer experience with Chatbot higher [27,30]. Also, the study by Zhang et al. (2021) suggested that people with higher levels of education were more likely to trust chatbots to provide accurate and helpful information [22]. While the study by Rudolph et al., (2023) indicated that Chatbot developers should consider the education level of their users when designing chatbots [1]. Users with higher education levels are more likely to use chatbots for complex tasks, while users with lower education levels are more likely to use chatbots for simple tasks. This may be because people with higher education levels are more likely to be familiar with artificial intelligence and are more likely to trust a chatbot to provide accurate and helpful information.

## 5 Conclusions

This section of the study focuses on its contributions to both the academic and applied fields of the topic of ChatGPT impact on the relationship between customer experience and overall satisfaction in digital marketing, also it includes the implications for future research.

### 5.1 Reflections on academic and applied fields

Current study offers valuable contributions for organizations considering incorporating ChatGPT into their digital marketing efforts. When deploying ChatGPT to improve customer experience, businesses should consider their consumers' age, education level, and familiarity & comfort with technology, as well as the types of their business. Businesses may optimize the impact of ChatGPT on the customer experience and improve their entire digital marketing strategies by customizing it to the individual demands and features of their consumers and business. It is expected that this study will be very useful for both academic and practical fields. In the academic field, it provides valuable insights into the potential of ChatGPT to improve customer experience in digital marketing. This information can be used to inform future research on ChatGPT and its impact on customer experience. In the practical field, this study can help businesses to make informed decisions about whether to use ChatGPT in their digital marketing efforts. The study's findings can also help businesses to optimize the impact of ChatGPT on customer experience by customizing it to the specific needs of their customers and business.

This study is likely to provide several advantages. For starters, it will give a better understanding of ChatGPT's influence on the customer experience in digital marketing. Second, it will determine whether there are any variances in this impact across different business types. Finally, it will help to design more effective digital marketing techniques that can improve the client experience. Fourth, it will provide valuable information for businesses that utilize ChatGPT to connect with consumers, allowing them to enhance their services and strengthen customer relationships. Overall, the conclusions of this study will be beneficial to firms, marketers, and scholars working in the field of digital marketing.

### 5.2 Implications for future directions

This study provides valuable insights into the potential of ChatGPT to improve customer experience in digital marketing. However, there are still many unanswered questions about the impact of ChatGPT on customer experience. Future research is needed to explore these questions and to further our understanding of the potential of ChatGPT to revolutionize digital marketing. There are four main future directions of study: (1) Investigate the impact of ChatGPT on customer experience in different cultures and countries. (2) explore the use of ChatGPT in different digital marketing channels. (3) Study the long-term impact of ChatGPT on customer relationships. (4) Explore the ethical implications of using ChatGPT in digital marketing. Overall, the conclusions of this study provide valuable insights into the potential of ChatGPT to improve customer experience in digital marketing. However, there are still many unanswered questions about the impact of ChatGPT on customer experience. Future research is needed to explore these questions and to further our understanding of the potential of ChatGPT to revolutionize digital marketing.

## 6 Limitations and future research

There are some limitations in the current study that should be addressed in future research. First, future research might utilize a representative sample to improve the study's external validity. Second, the study relied on self-reported data, which is susceptible to response bias. To give a more thorough assessment of the influence of ChatGPT on customer experience, a future study might add objective measurements of consumer experience, such as behavioral data and physiological markers. Third, the study concentrated on the influence of ChatGPT on customer experience rather than other marketing outcomes such as sales and brand loyalty. Further study might investigate these findings to offer a more thorough review of ChatGPT's performance in digital marketing. In addition to resolving these constraints, further study should investigate other elements that may influence the link between ChatGPT and the customer experience. Finally, the reproducibility of the article could be significantly increased and expanded in the following ways: (1) The findings could be sent to marketing companies to be tested on a larger data set and given to a larger number of people. (2) The findings could be used as a compass to develop a new questionnaire focusing on consumer behavior through the interaction of digital marketing with ChatGPT.

## Author contribution statement

Osama Ahmed Abdelkader; Conceived and designed the experiments; Performed the experiments; Analyzed and interpreted the data; Contributed reagents, materials, analysis tools or data; Wrote the paper.

## Data availability statement

Data included in article/supp. material/referenced in article.

## Additional information

No additional information is available for this paper.

## Declaration of generative AI and AI-assisted technologies in the writing process

During the preparation of this work the author used Bard Google [32] in brainstorming and generate ideas, and Quill Bot [33] to proofread and revise the grammar of the manuscript. After using this tool/service, the author reviewed and edited the content as needed and takes full responsibility for the content of the publication.

## Declaration of competing interest

The authors declare that they have no known competing financial interests or personal relationships that could have appeared to influence the work reported in this paper.
